# Patch clamp studies on TRPV4-dependent hemichannel activation in lens epithelium

**DOI:** 10.3389/fphar.2023.1101498

**Published:** 2023-02-24

**Authors:** Jose F. Ek-Vitorin, Mohammad Shahidullah, Joaquin E. Lopez Rosales, Nicholas A. Delamere

**Affiliations:** ^1^ Department of Physiology, University of Arizona, Tucson, AZ, United States; ^2^ Department of Ophthalmology and Vision Science, University of Arizona, Tucson, AZ, United States

**Keywords:** lens, TRPV4, connexin, hemichannels, patch-clamp, conductance, depolarization, K+ channels

## Abstract

ATP release from the lens *via* hemichannels has been explained as a response to TRPV4 activation when the lens is subjected to osmotic swelling. To explore the apparent linkage between TRPV4 activation and connexin hemichannel opening we performed patch-clamp recordings on cultured mouse lens epithelial cells exposed to the TRPV4 agonist GSK1016790A (GSK) in the presence or absence of the TRPV4 antagonist HC067047 (HC). GSK was found to cause a fast, variable and generally large non-selective increase of whole cell membrane conductance evident as a larger membrane current (Im) over a wide voltage range. The response was prevented by HC. The GSK-induced Im increase was proportionally larger at negative voltages and coincided with fast depolarization and the simultaneous disappearance of an outward current, likely a K^+^ current. The presence of this outward current in control conditions appeared to be a reliable predictor of a cell’s response to GSK treatment. In some studies, recordings were obtained from single cells by combining cell-attached and whole-cell patch clamp configurations. This approach revealed events with a channel conductance 180–270 pS following GSK application through the patch pipette on the cell-attached side. The findings are consistent with TRPV4-dependent opening of Cx43 hemichannels.

## 1 Introduction

The eye lens expresses abundant connexin (Cx) proteins. Cells of the anterior epithelial layer express Cx43 and Cx50 (DeRosa et al., 2009; Berthoud et al., 2013; Berthoud et al., 2014). These are the most metabolically active cells in the lens, but they comprise a vanishingly small part of its bulk (Delamere and Tamiya, 2004; Chauss et al., 2014; Zahraei et al., 2022). Mostly, the lens is a tightly packed mass of elongated fiber cells. The mature fibers, which lose organelles and nuclei as they develop, contain Cx50 and Cx46 (Biswas et al., 2010). The connexin protein family is best known for making cell-to-cell gap junction channels that allow electrical coupling and the exchange of metabolites, second messengers and ions between neighboring cells (Ek-Vitorin and Burt, 2013). In the lens, connexins are essential for ion and water homeostasis of the fibers that depend on transport mechanisms located in the epithelial monolayer on just the anterior surface (Delamere and Tamiya, 2004; Chauss et al., 2014; Zahraei et al., 2022). Gap junctions are formed by hexameric Cx arrangements (connexons) on each side of the virtual gap between contacting cell membranes, that dock and open to form intercellular pathways. The undocked connexons (or hemichannels, HCh) of several Cxs, including Cx43, can open under certain conditions (Contreras, 2003; Srinivas et al., 2005; Tong and Ebihara, 2006; Ebihara et al., 2011; Beyer and Berthoud, 2014; Ebihara et al., 2014; Liu et al., 2020), temporarily connecting the intracellular and extracellular spaces and allowing signals to flow on both directions. Cx43 is widely distributed in the organism, and has been extensively studied; Cx43 gap junctions are the most permeable to molecular ions, and are well regulated by many cytoplasmic agents, including phosphorylation (Ek-Vitorin and Burt, 2013). In recent years Transient Receptor Potential Vanilloid channels 1 and 4 (TRPV1 and TRPV4) have been detected in lens cells (Shahidullah et al., 2012; Delamere et al., 2016; Nakazawa et al., 2019). Various lines of evidence indicate TRPV1 and TRPV4 enable the lens epithelium to sense mechanical stimuli such as osmotic shrinking and swelling and respond by changing transporter activity to compensate (Delamere et al., 2020; Delamere and Shahidullah, 2021). There are two separate feedback loops, one responding to hypoosmotic stimuli, the other to hyperosmotic stimuli. TRPV4 is operational in the feedback loop that responds to osmotic swelling (Shahidullah et al., 2020). TRPV4 activation, either by an osmotic stimulus, or stretch, or a small molecule agonist, triggers a series of steps that include an increase of cytoplasmic calcium ([Ca^2+^]_i_), ATP release, purinergic receptor activation, Src kinase activation and, ultimately, an increase of Na/K-ATPase activity (Shahidullah et al., 2015). The release of ATP *via* hemichannels has been proposed as a critical early step in this chain of events. The notion that hemichannel opening causes the observed ATP release is supported by studies on the entry of large molecules, such as propidium iodide, following TRPV4 activation (Shahidullah et al., 2011). Moreover, ATP release can be blocked by recognized Cx inhibitors. Thus, a specific functional link between TRPV4 activation and Cx hemichannel opening has been proposed (Shahidullah et al., 2012). In contrast, there is no evidence that hemichannel-like responses are induced by TRPV1 stimulation. Hemichannel opening is an interesting phenomenon and the discovery of its apparent link to TRPV4 was unexpected. The present study was carried out to find direct electrophysiological evidence of TRPV4-induced hemichannel opening in lens epithelial cells. We also wanted to establish a patch clamp approach that could be used to further study the mechanism in the lens, and to study other cells in which TRPV4 and hemichannels might interact in a similar manner.

## 2 Methods

### 2.1 Chemicals

TRPV4 agonist GSK1016790A (GSK) was purchased from Selleckchem (Houston, TX, Cat. #S6637). TRPV4 antagonist HC067047 (HC) was purchased from Sigma (Saint Louis, MO; Cat. # SML0143). Because TRPV4 can be activated by several stimuli, including heat and mechanical stress (Nilius et al., 2004), we avoided temperature changes and fast superfusion to study the separate effects of the chemicals. Thus, experiments were conducted at room temperature (22°C–24°C); agonist and antagonist were diluted in external solution and gently dripped onto the recording chamber for final concentrations of 10 nM GSK1016790A and 10 µM HC067047. For TRPV4 inhibition, cells were preincubated with HC067047 at room temperature for at least 30 min before the start of recordings.

### 2.2 Cells culture

Primary cultures of lens epithelial cells (LEC) were obtained from freshly isolated mouse lens obtained from 8 to 20 weeks old male or female adult mice (C57BL6/J) (Jackson Laboratory, Maine). Isolation of lenses and culture of epithelium were done according to our published methods (Shahidullah et al., 2012; Shahidullah et al., 2022). The use of animals was approved by the Institutional Animal Care and Use Committee (IACUC) of the University of Arizona. The approved protocol number for mouse lens experiments is 18–492. The lens is cleanly isolated by cutting the zonules that hold it in position between the aqueous and vitreous humor. It has no blood supply and no nerves and consequently the potential for contamination by non-lens cells is close to zero. The lens itself is formed by just two cell types: epithelium and fibers. The lens epithelial cells are firmly attached to the acellular capsule which envelops the lens. The capsule with the attached epithelium is easy to remove from the mass of fiber cells that make up the bulk of the lens structure. The epithelial cells are able to divide but fiber cells are not. This made it possible for us to isolate the capsule/epithelium and allow the cells to grow in primary culture. Previously we confirmed expression of E-cadherin and absence of N-cadherin, typical of epithelium, and expression of Cx43 which is expressed only in the epithelial cells. In brief, we removed the capsule/epithelium from four to six lenses and placed them on a culture dish (60 mm) in a CO_2_ incubator at 37°C. About 0.5 mL of complete culture medium was placed around the border of the dish to maintain humidity. After 30 min, 3–4 mL of complete medium was added to flood the dish and cover the capsule-epithelium explants. The complete medium was prepared using an Epithelial Cell Medium kit (Sciencell Research Laboratories, Carlsbad, CA): 500 mL Basal Epithelial Cell Medium, 10 mL Fetal Bovine Serum (FBS) and 5 mL of a mixture of penicillin and streptomycin. The medium was changed at day 3 and then on alternate days. When enough cells had grown out of the explants, which takes 7–8 days, the cells were trypsinized and propagated as follows. The medium was removed, and the cells washed 2x with Ca^2+^-free, Mg^2+^-free HBSS and then subjected to low speed shaking for 3 min in 4.0 mL of 0.25% Trypsin EDTA solution. An equal volume of a mixture of FBS and newborn calf serum (1:1) was added to neutralize the trypsin, then the cell suspension was centrifuged at 167 *g* for 10 min. The pellet was then resuspended in 4 mL of complete medium and seeded in 25 cm^2^ flask at a density of ∼10,000–15,000 cells/cm^2^. The medium was changed after 1 day, then on alternate days. Cells typically became confluent in 4–5 days and were propagated to the next passage. In the present study second to fifth passage cells were used.

### 2.3 Electrophysiology

Cells were plated on glass coverslips at very low density to obtain isolated single cells, incubated overnight in culture medium and used between 40 and 50 hs after plating. Coverslips were attached with Vaseline to the perforated bottom of 4–5 mL plastic chambers (35 mm cell culture dish caps) and placed on the stage of an upright microscope (BX50WI, Olympus) for patch-clamp work. Two discontinuous single-electrode voltage-clamp (DSEVC) amplifiers (SEC-05LX NPI, Germany) were used to simultaneously clamp the voltage and record membrane currents from two single cells in whole-cell voltage clamp (WCVC) mode, or from a single cell with one electrode in WCVC and the second in cell-attached voltage clamp (CAVC) mode. In some experiments, WC configuration was achieved but voltage clamp was not performed: instead, the resting membrane potential (Vm) was documented using the amplifiers’ bridge mode (BR) function. Cells were bathed in external solution (in mM: NaCl 140, KCl 4.7, CaCl_2_ 1.8, MgCl_2_ 1.2, Glucose 10, EGTA 0.1, HEPES 10) adjusted to pH = 7.2 and osmolarity 319–330 mOsm. Patch pipettes were filled with internal (in mM: KCl 138, MgCl_2_ 3, TEACl 9, CaCl_2_ 0.5, EGTA 9, Glucose 5, Na_2_ ATP 5, HEPES 9; adjusted to pH = 7.4 and osmolarity 315–319 mOsm) or external solution.

Pulsing protocols were as follows: “steady HP”, continuously voltage clamping at any holding potential (HP); “−70 to +80 mV”, from an HP = −70 mV, 600 ms, +80 mV square pulses applied every 10–30 s; “±100 mV ramps”, from and HP = −50 mV, voltage ramps from −100 to +100 mV (slope ∼32 mV per sec) applied every 10–30 s; “step or IV”, from HP = −70 mV, 600 ms square pulses from −80 to +80 mV in 20 mV steps, every 5.5 s; “±80 paired pulses”, from HP = 0 mV, 10 s pulses to −80 and +80 mV, preceded by 100 ms prepulses at ±10 mV, every 30 s; “long pulses”, HP at any given value, usually −80, +80 mV or 0 mV, held for variable periods >1 s. Recordings were acquired at 13 kHz and low pass filtered at 1 kHz. Channel recordings were further filtered (100–200 Hz) and decimated (10–50) for ease of display. Macroscopic Im was analyzed with Clampfit10 (Molecular Probes) and Excel (Microsoft). For single channel event amplitude, long traces were surveyed with the Histogram function of Clampfit10 to locate sections with discrete transitions, then transferred those sections to Excel to produce refined all-points histograms with bin sizes of 0.25–0.5 pS; traces and histograms were plotted with SigmaPlot (SPSS Inc.). When suitable, results are reported as average ±SEM.

## 3 Results

### 3.1 TRPV4 agonist GSK increased membrane conductance and caused depolarization

**FIGURE 1:**
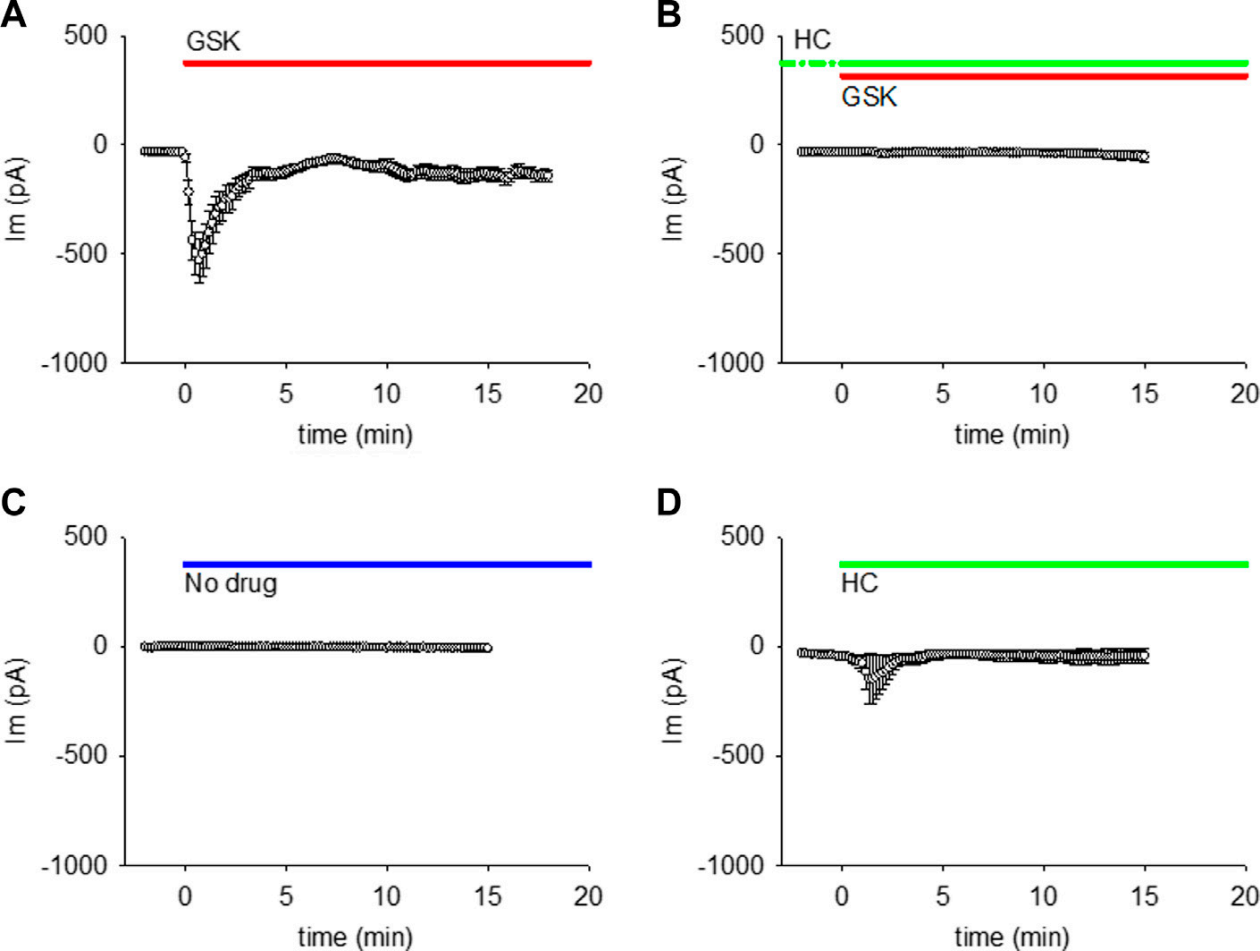
TRPV4 agonist GSK1016790A (GSK) increased the membrane conductance (gm) of lens epithelial cells, while TRPV4 antagonist HC067047 prevented this GSK-induced gm increase. **(A, B)** TRPV4 activation increased the WC Im at a holding potential of −50 mV (A, *n* = 27), while TRPV4 inhibition prevents that Im increase (B, n = 12). **(C, D)** WC Im at holding potential of −50 mV did not increase with external solution without drug (C, *n* = 7), while HC alone caused a small increase in one out of five cells (D, *n* = 5). For these and all akin figures, the lines on top of each plot indicate the application and continuous presence of the used drug.

When single lens epithelial cells were set at a steady holding potential of −50 mV by whole cell voltage clamp, the TRPV4 agonist GSK1016790A (GSK; 10 nM) increased membrane conductance (gm) as revealed by an increase in whole cell membrane current (WC Im) (Figure 1A). The amplitude and time frame of the gm increase was variable (Supplemental Figure S1), but generally large and fast (within the first 2 min of GSK application). The gm response reached a maximum (peak) at ∼40 s, then decreased to a plateau value larger than the initial baseline level for the rest of the experiment (up to 40 min). Incubation with the TRPV4 antagonist HC067047 (HC, 10 μM) for ≥30 min prevented the GSK-induced gm increase (Figure 1B). Because the effects of GSK and HC were similar in cells obtained from male and female mice (Suppl. Fig. 1), the results shown in Figure 1 and following figures were pooled. No large gm increase was seen when external solution without drug, or with HC alone were used (Figures 1C,D). However, one of the cells in Figure 1D responded to HC with a small, transient Im increase. This is consistent with the high variability of cells’ response, and with the possibility that HC has a minor agonistic effect.

**FIGURE 2:**
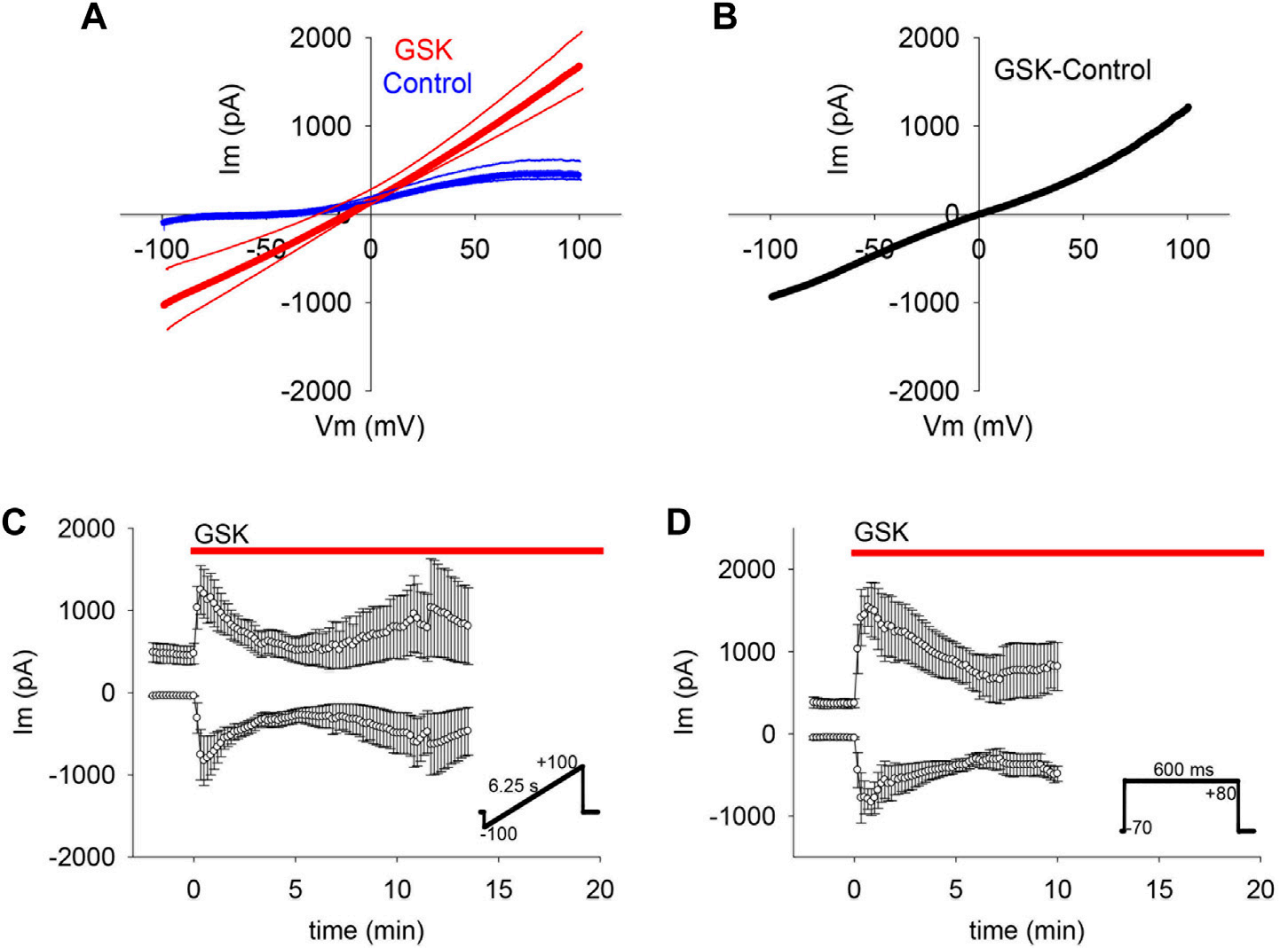
TRPV4 agonist GSK1016790A (GSK) increased gm on a wide voltage range. **(A–C)** Voltage ramps (inset in C) showed that TRPV4 activation increased WC Im in the range between −100 and + 100 mV (A, average of *n* = 7); for clarity, SEM is shown as thin lines accompanying each curve). The net effect of TRPV4 activation on gm was evinced by subtracting the control from the GSK treated curve (B). The mean WC Im between −100 and −60, and between +60 and +100 mV plotted in time show the persistent gm changes (C, average of *n* = 7). **(D)** Square pulses (inset) confirmed the TRPV4 associated WC Im increases at −70 and +80 mV (*n* = 6).

To determine whether the GSK-induced gm increase was limited to a particular voltage range or polarity (as would be expected from ion selective channels), or evident across a wider voltage range (as expected from non-selective pores), GSK treatment was performed while repeated ±100 mV voltage ramps were applied. WC Im responses recorded before and after GSK addition were plotted against voltage (Figure 2A and Supplementary Figure S2). In control conditions, Im was small at negative polarities and larger at positive polarities, gradually increasing to a plateau between +80 and +100 mV. This is consistent with lower channel activity at resting membrane potential than at depolarizing voltages that activate ionic outward currents (control traces Figure 2A and Supplementary Figure S2A). Immediately after GSK application, WC Im increased at both branches of the ramp, and the increase was proportionally larger at negative polarities. Subtraction of the average control from the GSK-treated ramps showed that the GSK-induced WC Im was near linear along the voltage range (Figure 2B).

In cells subjected to a voltage ramp, the means of the WC Im measured from −100 to −60, and from +60 to +100 mV were plotted against time (Figure 2C). The results showed that the GSK-induced gm increase was large at both polarities and confirmed a larger gm increase at the normal negative resting membrane potential. This is important because the proposed hemichannel opening in the lens almost certainly initiates at the normal membrane potential (Mandal et al., 2015). Hemichannel activity in lens cells at negative voltage was also observed in studies that employed square pulses alternating between a holding potential of −70 mV (resembling resting potential) and +80 mV (Figure 2D).

**FIGURE 3:**
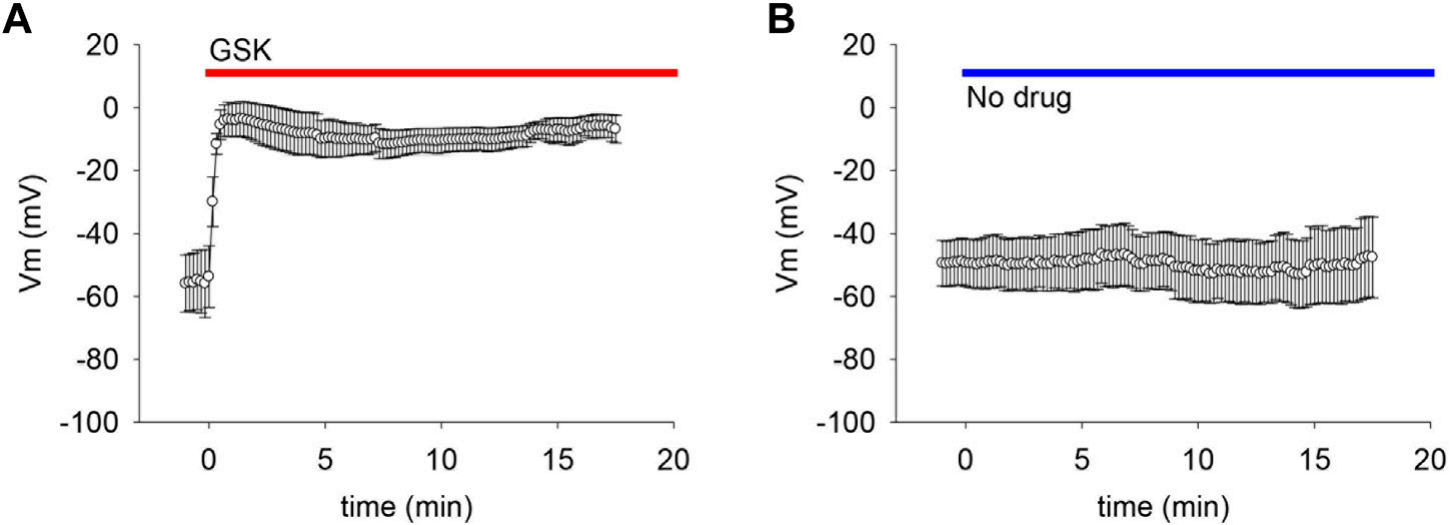
TRPV4 agonist GSK1016790A (GSK) caused depolarization of lens epithelial cells. **(A)** TRPV4 activation depolarized the resting membrane potential (Vm) of LEC (*n* = 6). **(B)** Dripping external solutions without drugs did not disturb Vm (*n* = 9).

If the observed gm increase was caused by the opening of non-selective pores like hemichannels, we reasoned that it would be likely to collapse the negative resting membrane potential. To explore this possibility, membrane potential was monitored while applying GSK. The TRPV4 agonist caused a sharp and long-lasting depolarization to values near 0 mV (Figure 3).

### 3.2 TRPV4 agonist GSK decreased outward transient currents

**FIGURE 4:**
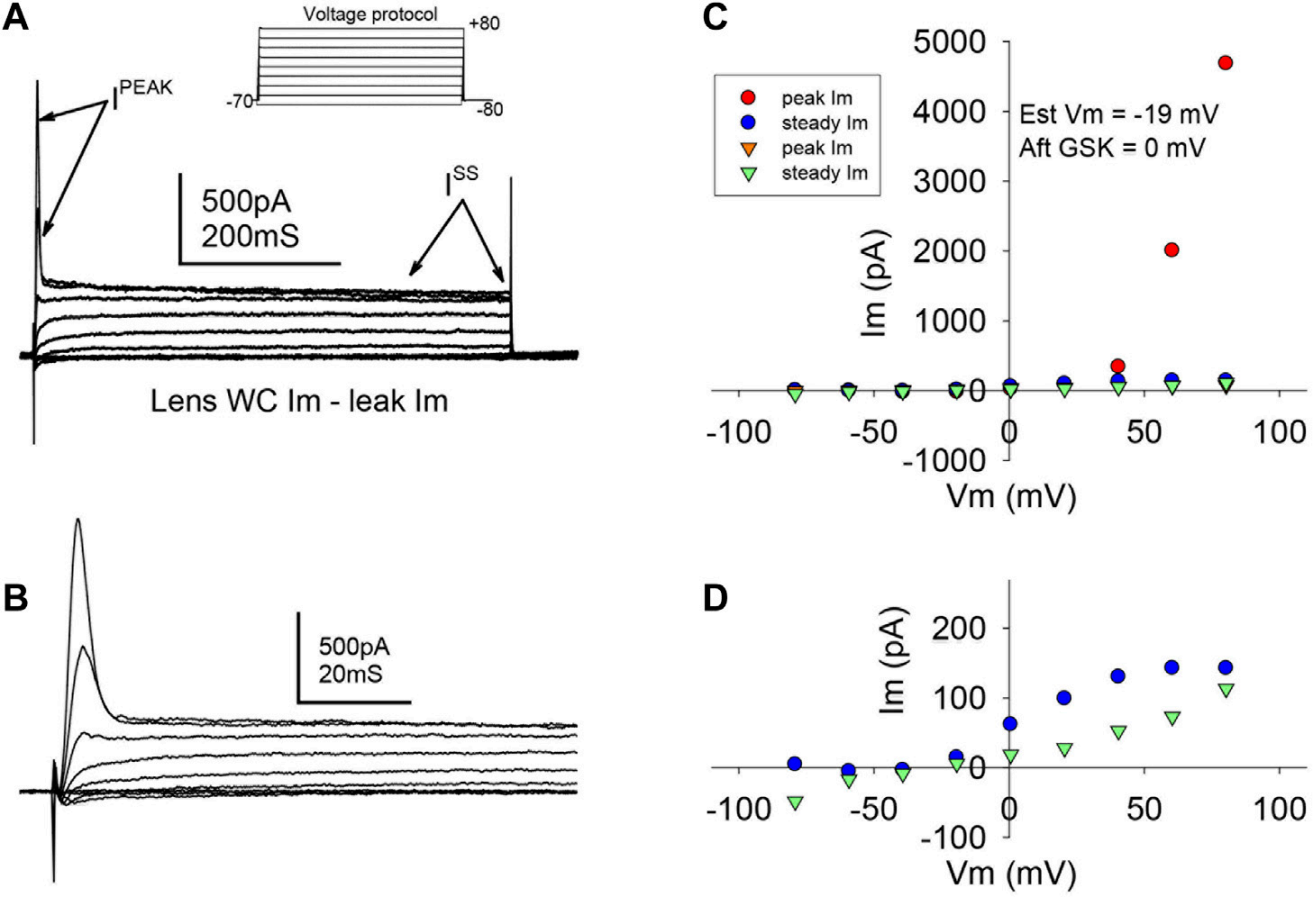
TRPV4 agonist GSK1016790A abolished a transient outward current. **(A,B)** Typical ionic current responses from single LEC in control conditions (A), elicited by a voltage protocol (inset) designed to explore K^+^ outward currents (See Methods, “step or IV” protocol, and Results for further description). A fast transient (I^peak^) and a slower steady state (I^ss^) current were evident. A close-up of I^peak^ is shown in (B). **(C)** Typical IV curve illustrating I^peak^ and I^ss^ before (circles) and after (downward triangles) TRPV4 activation. **(D)** Magnified amplitude section from C showing only I^ss^. Notice that I^peak^ is abolished, while I^ss^ is partially reduced. The resting potential was estimated (Est Vm) as the voltage needed to set the injected current at 0 pA in WCVC, and it decreased to 0 mV after TRPV4 activation.

**FIGURE 5:**
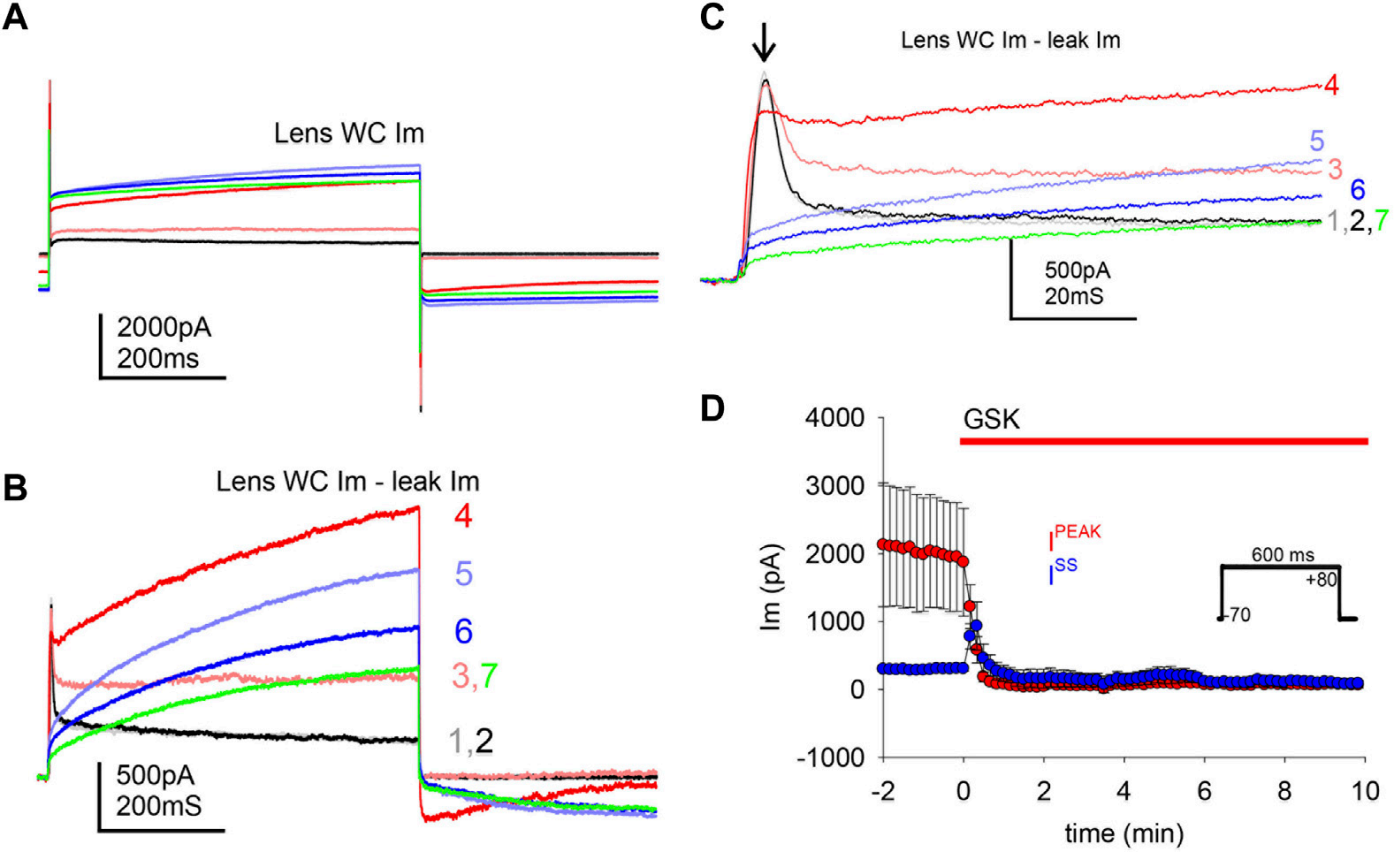
TRPV4 agonist GSK1016790A abolished a transient outward current. TRPV4 agonist GSK1016790A caused an increase of the whole cell gm, and a decrease of outward ionic currents. **(A)** Illustration of the large WC Im increase induced by TRPV4 activation while applying square pulses (inset in D). **(B–C)** Ionic outward currents evinced by subtraction of putative leak current from traces in A (color coded to indicate correspondence to the traces in A and numbered to indicate pulse order every 10 s); a closer look of the transient I^peak^ is shown in C (arrow). GSK1016790A was applied at the second pulse. **(D)** Average of transient peak (I^peak^, maximum) and steady state (I^ss^, last 100 ms) outward currents, during GSK application (*n* = 7).

Studies were carried out to search for outward currents that could represent flux through K^+^ channels during our pulsing protocols. In part, this was accomplished by subtracting leak currents. Cells subjected to a protocol that shifted holding potential in steps from −80 to +80 mV showed a steady outward current (I^ss^) appearing at a holding potential of −20 mV and growing with each step until a plateau at values higher than +40 mV (Figure 4A). At a holding potential of +60 mV, a transient outward current (I^peak^) appeared and grew with the next steps (Figures 4A,B). IV curves before and after GSK (Figures 4C, D) showed that the TRPV4 agonist abolished I^peak^ and decreased I^ss^. Resting potential (Table 1) was determined as the voltage required to set I_m_ to zero in whole cell voltage clamp mode. To further explore the behavior of the outward currents, we treated cells with GSK while applying repeated −70 to +80 mV square pulses every 10 s. In control conditions, leak current subtraction showed the two outward currents, I^peak^ and I^ss^ (Figures 5A–C). Within 1 minute of GSK application, the WC I_m_ displayed a large increase (Figure 5A). Simultaneously, I^peak^ disappeared, while I^ss^ showed a slight transient increase followed by a decrease to values smaller than the initial baseline (Figure 5D). Together, these data suggest that blocking of K^+^ currents may contribute to depolarization of GSK-treated cells. Of note, it is commonly known that Na^+^ and Ca^2+^ channels would allow inward currents, while Cl^−^ channels typically open at very negative voltages.

**TABLE 1:** Resting membrane potential values estimated in whole cell voltage clamp, before and after the application of GSK1016790A.

Table 1		
Estimated resting membrane potential		
Condition	Control	GSK
Average	−43.85	−2.75
STDEV	17.51	1.08
N	20	8

**FIGURE 6:**
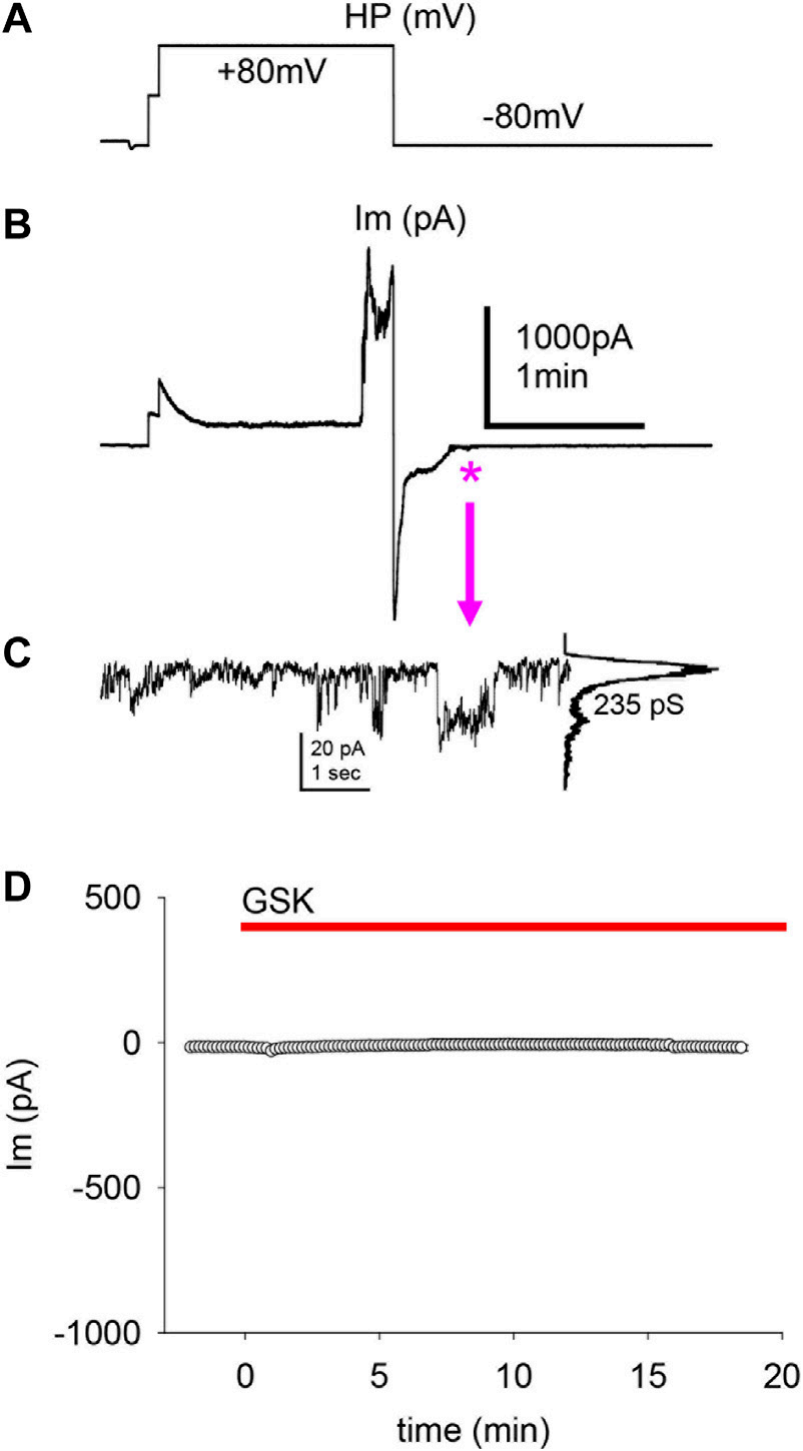
The transient peak Im was predictive of a cell’s response to depolarization and GSK1016790A treatment. **(A–B)** Applied voltages (A) and resultant WC Im (B) from single LEC with a large I^peak^ in control conditions (no GSK present). A large, spontaneous increase of WC Im developed after ∼1 min of sustained +80 mV pulse, and a fast return to initial Im levels ensued at a subsequent −80 mV pulse. The swiftness of the WC Im increase impairs the assessment of HCh-like step-wise transitions. **(C)** Transitions of conductance consistent with Cx43 HCh (235 pS) were seen on an enlargment of the Im recovery end (asterisk and arrow). This value is the channel conductance (*γ*) of the larger transitions: current amplitude difference between the baseline (large peak) and the maximal opening (small peak), shown in the all-points histogram (right of the trace) of the illustrated events, divided by the voltage gradient (γ = ΔIm/Vm). **(D)** Cells with a proportionally small I^peak^ or proportionally larger I^ss^ showed little or no response to GSK (average of *n* = 5).

The obliteration of a transient outward current by GSK suggests that the decrease of I^peak^ and the ensuing depolarization are significant. To examine this further, cells with a large I^peak^ and proportionally smaller I^ss^ in control conditions were held at −80 and +80 mV for long pulses. In Figures 6A,B, one of two single cells that were simultaneously recorded in the absence of GSK showed a spontaneous WC Im increase during an +80 mV pulse longer than 1 minute; repolarization to −80 mV was sufficient to quickly return WC Im to previous levels. In this experiment, a few ≥200 pS transitions were seen at the end of the recovery (Figure 6C). The second cell showed similar WC Im increase and decrease at +80 and −80 mV, respectively, but no distinct events were seen at recovery. Large WC Im increases at +80 mV and recoveries at −80 mV were seen often, but not always, in control conditions (data not shown). During the course of these studies, it became apparent that in cells with a substantial I^peak^ (smaller I^ss^), depolarization is sufficient to cause a large increase in gm. Next, cells with proportionally small I^peak^ (or larger I^ss^) were found and clamped at a holding potential of −50 mV. These cells did not show significant Im increases with GSK treatment (Figure 6D). The findings suggest that the I^peak^ is necessary for the GSK-induced Im increase, and depolarization is essential for the effects of TRPV4 activation.

### 3.3 TRPV4-associated hemichannel-like transitions

**FIGURE 7:**
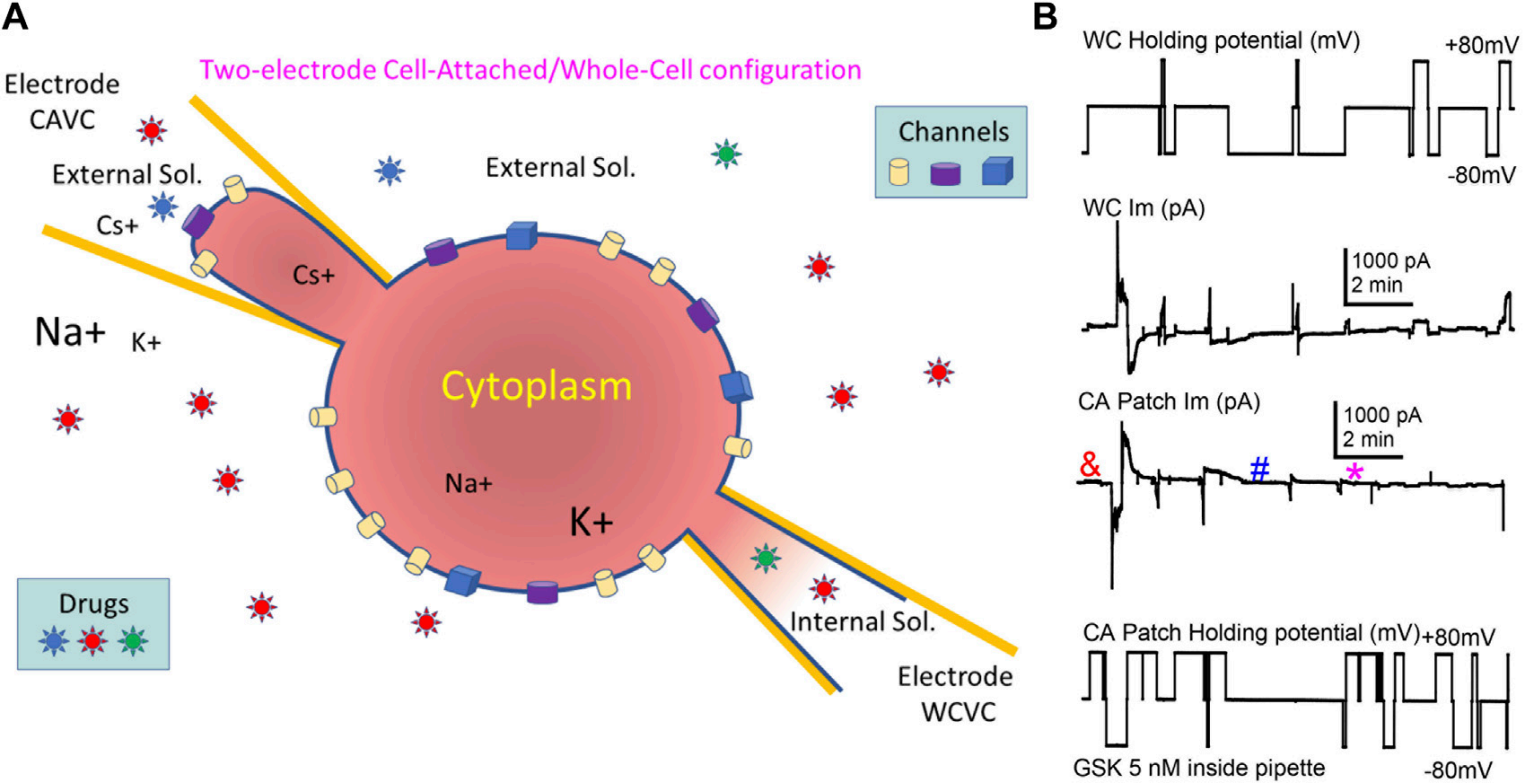
The combination of Cell-Attached and Whole-Cell (CA/WC) modes in a single cell enabled the recording of channels induced by GSK1016790A. **(A)** Illustration of CA/WC Voltage Clamp configuration in single cell. Voltage pulses can be applied on either side (intracellular or extracellular) of the attached patch, and Im is recorded from both sides as mirror images. Drugs can be applied intracellularly or extracellularly (in the bath or through the CA pipette), and channels partaking of a large Im increase in the whole cell can be documented. **(B)** Example of a WC/CA recording. Voltage applied to the WC (top) or the CA (bottom) side, and the corresponding induced currents (middle traces) are displayed. After achieving WCVC with one electrode filled with internal solutions, the cell was approached with a second electrode containing 5 nM TRPV4 agonist GSK1016790A diluted in external solution, and CAVC was quickly achieved. Pulses to ±80 mV were alternately applied to either side, while keeping the opposite at 0 mV. Notice that after a short delay (<1 min), a transient increase of gm (larger Im values) occurred that seem to reside on the membrane patch alone. Channel events were detected before (&) and after (# and *) the gm increase (see Figure 8).

**FIGURE 8:**
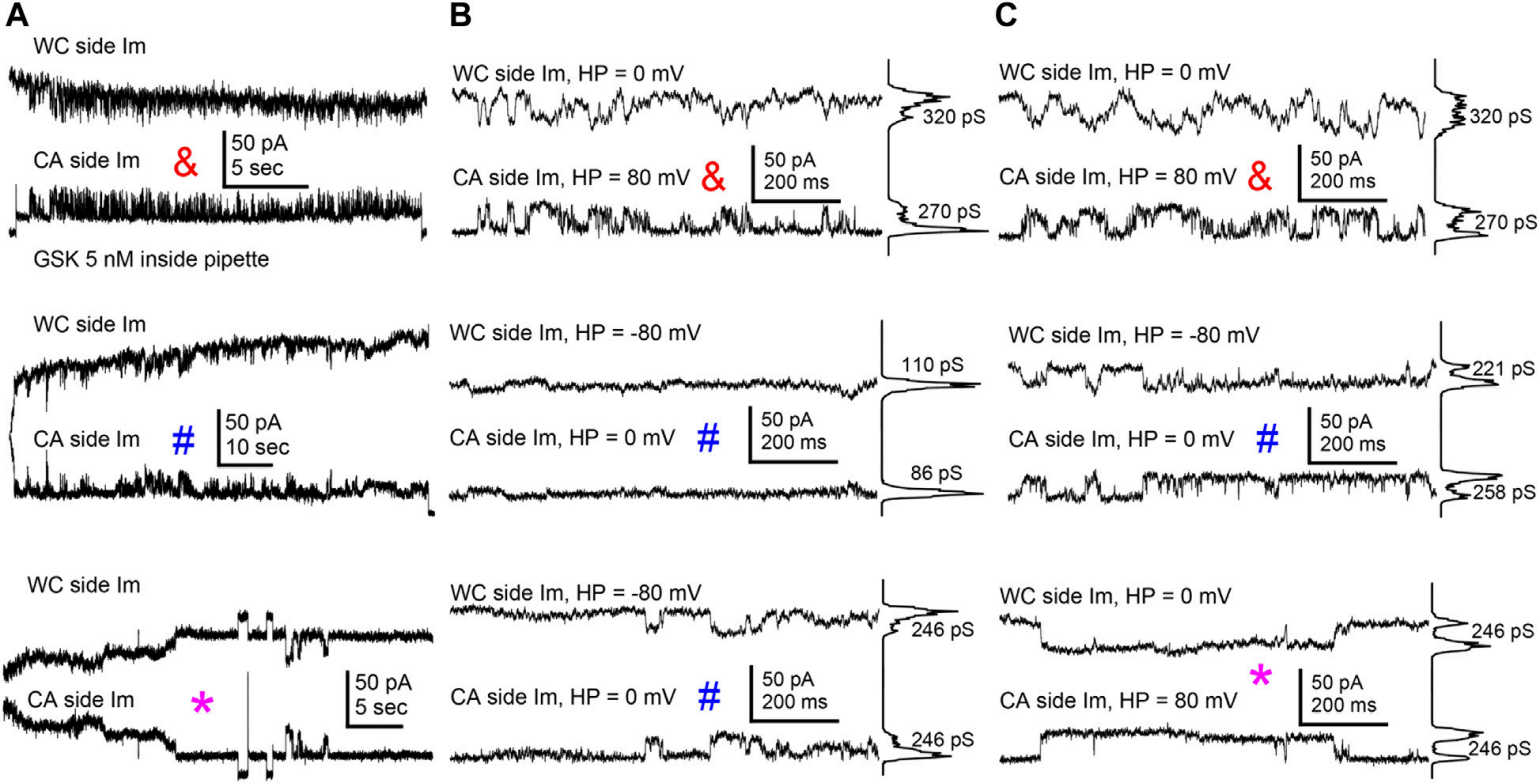
TRPV4 agonist GSK1016790A induced channel activity consistent with Cx43 HCh. **(A)** Long stretches of channel activity on the attached membrane patch recorded with the WC/CA combined configuration before (&) and after (# and *) the GSK1016790A-induced transient gm increase shown in previous figure (symbol and color coded as in Figure 7). **(B–C)** Expanded segments from the traces on A showed mirrored channel transitions compatible with Cx43 HCh. All-points histograms at the right of the shorter stretches indicate the most apparent transition conductance values. Transitions smaller than 100 pS (traces at the center of figure), compatible with reported amplitude of TRPV4 channels, were also seen.

In mammalian cells with low Im levels, Cx43 HCh transitions (∼220 pS) appear distinctly at voltages > +60 mV (Contreras, 2003) similar to the transitions shown in lens cells (Figure 6C). However, at such positive voltages and in control conditions, lens cells displayed a high Im that became larger when TRPV4 was activated by GSK (Figures 2, 5A), making the detection of single HCh transitions fortuitous at best and unfeasible in most cases. To address this issue, we used two electrodes on a single cell, one in whole cell voltage clamp and the second in cell-attached voltage clamp configuration (Figure 7A). With this approach we were able to record WC Im and simultaneously monitor a fraction of that current as channel events in a membrane patch (patch Im). As the voltage is controlled on both sides of the patch, this approach renders accurate measurements of the transmembrane voltage gradients and of the channel amplitudes. We applied GSK from inside the cell-attached pipette filled with extracellular solution. Concentrations of GSK ≥10 nM caused large increases of conductance of the attached membrane (patch gm) and eventual loss of cell integrity (not shown). The patch gm increase was lower in magnitude with lower concentrations of GSK (5 and 2 nM) in the cell-attached pipette. Using this approach, channel transitions were documented before and after the patch gm increase (Figure 7B). Recordings from the two sides of the patch, intracellular labelled WC, extracellular labelled cell-attached (CA), appear as mirror images (Figure 8). Because the CA mode records from a small membrane area, the current transitions are clearly defined and yield better amplitude measurements. Notice that at the CA patch, the intracellular voltage value for the channels present in the membrane is the opposite of the holding potential. Short segments of recordings were expanded to examine current transitions (Figures 8B, C). They show at least two event amplitude ranges: 50–90 pS, which falls in the range of the reported values for TRPV4 channels (Earley, 2010), and 180–270 pS, a range consistent with Cx43 but not Cx50 hemichannels. Interestingly, in the example shown, events appear in a negatively polarized membrane patch, demonstrating that HCh can open at a negative resting potential.

## 4 Discussion

Conductive ATP release requires ATP-permeable channels because the size and negative charge of ATP mean it is otherwise unable to diffuse across the lipid bilayer (Taruno, 2018). In the lens ATP release occurs following TRPV4 activation and there is indirect evidence that the mechanism involves TRPV4-dependent Cx hemichannel or pannexin opening (Shahidullah et al., 2012). Being cation selective, TRPV4 channels themselves are not available to ATP. Here, we determined to find electrophysiological evidence that activation of cation-selective TRPV4 channels in LEC induces the opening of non-charge selective pores compatible with a Cx HCh. We found that TRPV4 activation increased gm with a quasilinear I/V relationship in the ±100 mV range, and caused fast depolarization of the resting potential, suggesting the opening of non-selective pores. The responses to the TRPV4 agonist GSK were prevented by the TRPV4 antagonist HC. Using a combination of two patch-clamp configurations in a single cell, we observed channel activity compatible with Cx43 hemichannels.

TRPV4 activation led to prolonged lens cell depolarization. Broadly speaking, depolarization can occur by two mechanisms. These mechanisms have opposite effects on gm. A decrease of K^+^ channel activity, causes depolarization and would lower gm. This includes the effects of K^+^ channel blockers like Cs^+^ and TEA^+^. An increase in the activity of channels with an equilibrium potential near or above 0 mV would also cause depolarization but would raise gm. This includes activation of Na^+^, Ca^2+^ and non-selective channels like TRPV4. Because Cx43 hemichannels are probably non-charge selective (Heyman and Burt, 2008; Heyman et al., 2009), their opening would also cause depolarization and increase gm (Contreras, 2003; Lillo et al., 2019). This is consistent with the lens cell response observed in our experiments. Like in lens cells, TRPV4 activation was found to cause depolarization of endothelial cells from lymphatic collecting tubes (Behringer et al., 2017) as well as other cell types (Shibasaki et al., 2007; Rakers et al., 2017; Mundt et al., 2018). It should be noted, however, that activation of TRPV4 channels causes hyperpolarization in other cells (Earley, 2011; Netti et al., 2017). Because TRPV4 activation allows Ca^2+^entry, cells that express Ca^2+^-activated K^+^ channels can display GSK-induced hyperpolarization as was observed in endothelial cells from resistance arteries (Earley, 2011; Zhang and Gutterman, 2011; Sonkusare et al., 2012; Filosa et al., 2013).

We examined the presence of K^+^-like outward currents and found that lens cells in control conditions display one transient and one slower outward current that are reduced by GSK exposure. The presence of the transient outward current in control conditions appeared to be a predictor of a cell’s response to GSK treatment. This transient current was rapidly eliminated by TRPV4 activation with GSK. It is noteworthy that the abrupt increase of WC Im at a holding potential of −50 mV, the disappearance of I^peak^ and the fast phase of depolarization all took place simultaneously. Possibly, the initial depolarization upon GSK exposure was due to the combined effect of increasing intracellular Ca^2+^, decreasing I^peak^ possibly by K^+^ channel closure, and HCh opening. The depolarization would then be maintained by both continuous inhibition of K^+^ currents along with continued hemichannel opening. Sustained hemichannel opening is consistent with the increase of gm as well as previously reported persistent increase of cytoplasmic calcium concentration. The timeframe of appearance of HCh-like events recorded with the combined WC/CA technique aligns with this possibility.

Understanding that lens epithelial cells express both Cx43 and Cx50 (DeRosa et al., 2009; Berthoud et al., 2013; Berthoud et al., 2014) we asked whether the patch clamp responses shed light on the identity of the hemichannels that open when TRPV4 is activated. The homomeric, homotypic gap junction channels formed by Cx43 and Cx50 can be characterized by their conductances and permselectivities. Thus, Cx50 has a channel conductance >200 pS (Srinivas et al., 1999), while Cx43, ≤120 pS (Fishman et al., 1991; Veenstra et al., 1992). Therefore, if TRPV4 activation opens Cx43 hemichannels, GSK would be expected to induce the appearance of hemichannel transitions ∼200 pS (twice the conductance of Cx43 gap junctions), rather than the larger, >400 pS reported for Cx50 HCh (Srinivas et al., 2005). Recordings from GSK-treated LEC in WC/CA configuration showed channel events of amplitude near those of Cx43 hemichannels.

We are unable to explain why the present patch clamp studies point to TRPV4-dependent Cx43 hemichannel opening while earlier studies on intact wild type and Cx50 −/− mouse lenses identified the TRPV4-dependent hemichannels as Cx50 (Delamere et al., 2020). Responses may be different in isolated cells that have poorly defined apical-basolateral polarity and lack normal gap junction coupling to fibers. Despite being electrically “smaller”, Cx43 is the more permeable to molecular ions and displays no charge selectivity (Heyman et al., 2009; Ek Vitorin et al., 2016) while Cx50 is permeable to Ca^2+^ but impermeable to the negatively charged inositol-1,4,5-triphosphate (IP^3^) molecule (Valiunas and White, 2020). Assuming that the permselective properties of connexons in gap junctions are similar in undocked membrane hemichannels, one might expect that negatively charged ATP will permeate Cx43 better than the slightly cation-selective Cx50 (Slavi et al., 2014). Panx1 is expressed in lens epithelial cells (Dvoriantchikova et al., 2006). Panx1 is known to form large conductance (400–500 pS) non-selective channels (Bao et al., 2004; Locovei et al., 2006; Locovei et al., 2007). The possible contribution of pannexin opening remains to be determined.

Although Cx43 HCh tend to open at high positive potentials (Contreras, 2003), we observed hemichannel opening at negative voltage (−80 mV). A few studies have reported similar observations, for instance, in astrocytes treated with pro-inflammatory cytokines (Retamal et al., 2007). Cytokines lowered junctional coupling and increased Cx43 hemichannel activity, leading to increased glucose uptake but decreased intercellular diffusion. Hemichannels also were found to open at negative membrane potential in astrocytes subjected to hypoxic preconditioning that induced the release of ATP, which was catabolized to the neuroprotective adenosine that accumulated in the extracellular space (Lin et al., 2008). Of note, ATP is more likely to leave the cell when the membrane potential is negative but in normal conditions hemichannels are closed (Kang et al., 2008). Thus, it might be that TRPV4 activation, or exposure to proinflammatory cytokines, or hypoxic preconditioning each may change the normal gating of hemichannels, making them more likely to open at a negative voltage. Recently, it was reported that in mouse cardiac myocytes Cx43 and ryanodine receptors (RyRs) are in close apposition, and stimulation of RyRs with caffeine causes Cx43 hemichannel openings at negative (−70 mV) potentials (Lissoni et al., 2021). These HCh openings were fast and flickering (∼9 ms events) and required both increased [Ca^2+^]_i_ and RyRs stimulation. At positive voltages the hemichannel openings were longer and did not require RyRs stimulation (Lissoni et al., 2021). In the present study, lens cell recordings showed abundant and long (>100 ms) hemichannel opening events at negative potentials. It is possible that the population of hemichannels that open at positive voltage is different from those that open at the normal, negative resting potential.

Lastly, the current study represents the early stage of our patch clamp exploration of channel activity in lens epithelial cells and there are many questions still to be addressed regarding the role of Cx50, pannexins, K^+^, Na^+^ and Cl^−^ channels and other TRP channels. It is too early to propose therapeutic implications, and yet we feel inclined to think about the possibility that mishandling of Ca^2+^ by a dysfunctional TRPV4-Cx43 HCh axis may have a role in the genesis of certain chronic conditions, like cataracts.

## 5 Conclusion

In lens epithelial cells, agonist stimulation of TRPV4 channels with GSK1016790A simultaneously increases membrane conductance, decreases a transient outward current, and causes a fast and sustained depolarization. The increase of membrane conductance is prevented by pre-incubation with the TRPV4 antagonist HC067047. This increase of membrane conductance can be partially explained by the opening of channels with a conductance amplitude consistent with Cx43 hemichannels. While the initial depolarization could be due to the combined effect of increased cation entry through TRPV4 channels and inhibition of K^+^ currents, the opening of Cx43 hemichannels probably underlies the persistent depolarization. The combined whole-cell and cell-attached patch clamp strategy employed in this study can be used to examine channel activity in cells that have corresponding changes in whole-cell conductance.

## Data Availability

The data repository can be found at https://arizona.box.com/s/33jz95xz7k0tvetkyhoyjap86v1ju362.

## References

[B1] Bao L. Locovei S. Dahl G. (2004). Pannexin membrane channels are mechanosensitive conduits for ATP. FEBS Lett. 572 (1-3), 65–68. 10.1016/j.febslet.2004.07.009 15304325

[B2] Behringer E. J. Scallan J. P. Jafarnejad M. Castorena-Gonzalez J. A. Zawieja S. D. Moore J. E. Jr. (2017). Calcium and electrical dynamics in lymphatic endothelium. J. Physiol. 595 (24), 7347–7368. 10.1113/JP274842 28994159 PMC5730853

[B3] Berthoud V. M. Minogue P. J. Osmolak P. Snabb J. I. Beyer E. C. (2014). Roles and regulation of lens epithelial cell connexins. FEBS Lett. 588 (8), 1297–1303. 10.1016/j.febslet.2013.12.024 24434541 PMC3992928

[B4] Berthoud V. M. Minogue P. J. Yu H. Schroeder R. Snabb J. I. Beyer E. C. (2013). Connexin50D47A decreases levels of fiber cell connexins and impairs lens fiber cell differentiation. Invest. Ophthalmol. Vis. Sci. 54 (12), 7614–7622. 10.1167/iovs.13-13188 24204043 PMC3835270

[B5] Beyer E. C. Berthoud V. M. (2014). Connexin hemichannels in the lens. Front. physiology 5, 20. 10.3389/fphys.2014.00020 PMC3920103 24575044

[B6] Biswas S. K. Lee J. E. Brako L. Jiang J. X. Lo W.-K. (2010). Gap junctions are selectively associated with interlocking ball-and-sockets but not protrusions in the lens. Mol. Vis. 16, 2328–2341. 21139982 PMC2994765

[B7] Chauss D. Basu S. Rajakaruna S. Ma Z. Gau V. Anastas S. (2014). Differentiation state-specific mitochondrial dynamic regulatory networks are revealed by global transcriptional analysis of the developing chicken lens. G3 (Bethesda) 4 (8), 1515–1527. 10.1534/g3.114.012120 24928582 PMC4132181

[B8] Contreras J. E. Saez J. C. Bukauskas F. F. Bennett M. V. L. (2003). Gating and regulation of connexin 43 (Cx43) hemichannels. Proc. Natl. Acad. Sci. U. S. A. 100 (20), 11388–11393. 10.1073/pnas.1434298100 13130072 PMC208767

[B9] Delamere N. A. Mandal A. Shahidullah M. (2016). The significance of TRPV4 channels and hemichannels in the lens and ciliary epithelium. J. Ocul. Pharmacol. Ther. 32 (8), 504–508. 10.1089/jop.2016.0054 27513167 PMC5069728

[B10] Delamere N. A. Shahidullah M. (2021). Ion transport regulation by TRPV4 and TRPV1 in lens and ciliary epithelium. Front. Physiol. 12, 834916. 10.3389/fphys.2021.834916 35173627 PMC8841554

[B11] Delamere N. A. Shahidullah M. Mathias R. T. Gao J. Sun X. Sellitto C. (2020). Signaling between TRPV1/TRPV4 and intracellular hydrostatic pressure in the mouse lens. Invest. Ophthalmol. Vis. Sci. 61 (6), 58. 10.1167/iovs.61.6.58 PMC7415899 32598448

[B12] Delamere N. A. Tamiya S. (2004). Expression, regulation and function of Na,K-ATPase in the lens. Prog. Retin Eye Res. 23 (6), 593–615. 10.1016/j.preteyeres.2004.06.003 15388076

[B13] DeRosa A. M. Mese G. Li L. Sellitto C. Brink P. R. Gong X. (2009). The cataract causing Cx50-S50P mutant inhibits Cx43 and intercellular communication in the lens epithelium. Exp. Cell Res. 315 (6), 1063–1075. 10.1016/j.yexcr.2009.01.017 19331825 PMC2670955

[B14] Dvoriantchikova G. Ivanov D. Pestova A. Shestopalov V. (2006). Molecular characterization of pannexins in the lens. Mol. Vis. 12, 1417–1426. 17149368

[B15] Earley S. (2011). Endothelium-dependent cerebral artery dilation mediated by transient receptor potential and Ca2+-activated K+ channels. J. Cardiovasc. Pharmacol. 57 (2), 148–153. 10.1097/FJC.0b013e3181f580d9 20729757

[B16] Earley S. (2010). Vanilloid and melastatin transient receptor potential channels in vascular smooth muscle. Microcirculation 17 (4), 237–249. 10.1111/j.1549-8719.2010.00026.x 20536737 PMC2925403

[B17] Ebihara L. Korzyukov Y. Kothari S. Tong J.-J. (2014). Cx46 hemichannels contribute to the sodium leak conductance in lens fiber cells. Am. J. Physiology Cell Physiology 306 (5), C506–C513. 10.1152/ajpcell.00353.2013 PMC4042622 24380846

[B18] Ebihara L. Tong J.-J. Vertel B. White T. W. Chen T.-L. (2011). Properties of connexin 46 hemichannels in dissociated lens fiber cells. Investigative Ophthalmol. Vis. Sci. 52 (2), 882–889. 10.1167/iovs.10-6200 PMC3053112 20861491

[B19] Ek Vitorin J. F. Pontifex T. K. Burt J. M. (2016). Determinants of Cx43 channel gating and permeation: The amino terminus. Biophys. J. 110 (1), 127–140. 10.1016/j.bpj.2015.10.054 26745416 PMC4805864

[B20] Ek-Vitorin J. F. Burt J. M. (2013). Structural basis for the selective permeability of channels made of communicating junction proteins. Biochim. Biophys. Acta 1828 (1), 51–68. 10.1016/j.bbamem.2012.02.003 22342665 PMC3389294

[B21] Filosa J. A. Yao X. Rath G. (2013). TRPV4 and the regulation of vascular tone. J. Cardiovasc. Pharmacol. 61 (2), 113–119. 10.1097/FJC.0b013e318279ba42 23107877 PMC3564998

[B22] Fishman G. I. Moreno A. P. Spray D. C. Leinwand L. A. (1991). Functional analysis of human cardiac gap junction channel mutants. Proc. Natl. Acad. Sci. - PNAS 88 (9), 3525–3529. 10.1073/pnas.88.9.3525 1850831 PMC51484

[B23] Heyman N. S. Burt J. M. (2008). Hindered diffusion through an aqueous pore describes invariant dye selectivity of Cx43 junctions. Biophys. J. 94 (3), 840–854. 10.1529/biophysj.107.115634 17921206 PMC2186237

[B24] Heyman N. S. Kurjiaka D. T. Ek Vitorin J. F. Burt J. M. (2009). Regulation of gap junctional charge selectivity in cells coexpressing connexin 40 and connexin 43. Am. J. Physiol. Heart Circ. Physiol. 297 (1), H450–H459. 10.1152/ajpheart.00287.2009 19465552 PMC2711728

[B25] Kang J. Kang N. Lovatt D. Torres A. Zhao Z. Lin J. (2008). Connexin 43 hemichannels are permeable to ATP. J. Neurosci. 28 (18), 4702–4711. 10.1523/JNEUROSCI.5048-07.2008 18448647 PMC3638995

[B26] Lillo M. A. Himelman E. Shirokova N. Xie L. H. Fraidenraich D. Contreras J. E. (2019). S-nitrosylation of connexin43 hemichannels elicits cardiac stress-induced arrhythmias in Duchenne muscular dystrophy mice. JCI Insight 4 (24), e130091. 10.1172/jci.insight.130091 31751316 PMC6975272

[B27] Lin J. H. Lou N. Kang N. Takano T. Hu F. Han X. (2008). A central role of connexin 43 in hypoxic preconditioning. J. Neurosci. 28 (3), 681–695. 10.1523/JNEUROSCI.3827-07.2008 18199768 PMC6670356

[B28] Lissoni A. Hulpiau P. Martins-Marques T. Wang N. Bultynck G. Schulz R. (2021). RyR2 regulates Cx43 hemichannel intracellular Ca2+-dependent activation in cardiomyocytes. Cardiovasc Res. 117 (1), 123–136. 10.1093/cvr/cvz340 31841141

[B29] Liu J. Riquelme M. A. Li Z. Li Y. Tong Y. Quan Y. (2020). Mechanosensitive collaboration between integrins and connexins allows nutrient and antioxidant transport into the lens. J. Cell Biol. 219 (12), e202002154. 10.1083/jcb.202002154 33180092 PMC7668387

[B30] Locovei S. Bao L. Dahl G. (2006). Pannexin 1 in erythrocytes: Function without a gap. Proc. Natl. Acad. Sci. U. S. A. 103 (20), 7655–7659. 10.1073/pnas.0601037103 16682648 PMC1472500

[B31] Locovei S. Scemes E. Qiu F. Spray D. C. Dahl G. (2007). Pannexin1 is part of the pore forming unit of the P2X(7) receptor death complex. FEBS Lett. 581 (3), 483–488. 10.1016/j.febslet.2006.12.056 17240370 PMC1868681

[B32] Mandal A. Shahidullah M. Delamere N. A. (2015). Calcium entry via connexin hemichannels in lens epithelium. Exp. Eye Res. 132, 52–58. 10.1016/j.exer.2015.01.012 25597520 PMC4352408

[B33] Mundt N. Spehr M. Lishko P. V. (2018). TRPV4 is the temperature-sensitive ion channel of human sperm. eLife 7, e35853. 10.7554/eLife.35853 29963982 PMC6051745

[B34] Nakazawa Y. Donaldson P. J. Petrova R. S. (2019). Verification and spatial mapping of TRPV1 and TRPV4 expression in the embryonic and adult mouse lens. Exp. Eye Res. 186, 107707. 10.1016/j.exer.2019.107707 31229503 PMC7441740

[B35] Netti V. Fernández J. Kalstein M. Pizzoni A. Di Giusto G. Rivarola V. (2017). TRPV4 contributes to resting membrane potential in retinal müller cells: Implications in cell volume regulation. J. Cell. Biochem. 118 (8), 2302–2313. 10.1002/jcb.25884 28098409

[B36] Nilius B. Vriens J. Prenen J. Droogmans G. Voets T. (2004). TRPV4 calcium entry channel: A paradigm for gating diversity. Am. J. Physiology - Cell Physiology 286 (2), 195–205. 10.1152/ajpcell.00365.2003 14707014

[B37] Rakers C. Schmid M. Petzold G. C. (2017). TRPV4 channels contribute to calcium transients in astrocytes and neurons during peri‐infarct depolarizations in a stroke model. Glia 65 (9), 1550–1561. 10.1002/glia.23183 28639721

[B38] Retamal M. A. Froger N. Palacios-Prado N. Ezan P. Saez P. J. Saez J. C. (2007). Cx43 hemichannels and gap junction channels in astrocytes are regulated oppositely by proinflammatory cytokines released from activated microglia. J. Neurosci. 27 (50), 13781–13792. 10.1523/JNEUROSCI.2042-07.2007 18077690 PMC6673621

[B39] Shahidullah M. Mandal A. Delamere N. A. (2015). Damage to lens fiber cells causes TRPV4-dependent Src family kinase activation in the epithelium. Exp. Eye Res. 140, 85–93. 10.1016/j.exer.2015.08.013 26318609 PMC4763713

[B40] Shahidullah M. Mandal A. Delamere N. A. (2011). Hyposmotic stress causes ATP release and stimulates Na,K-ATPase activity in porcine lens. Investigative Ophthalmol. Vis. Sci. 52 (14), 3411. 10.1002/jcp.22858 21618533

[B41] Shahidullah M. Mandal A. Delamere N. A. (2012). TRPV4 in porcine lens epithelium regulates hemichannel-mediated ATP release and Na-K-ATPase activity. Am. J. Physiol. Cell Physiol. 302 (12), C1751–C1761. 10.1152/ajpcell.00010.2012 22492652 PMC3378078

[B42] Shahidullah M. Mandal A. Mathias R. T. Gao J. Križaj D. Redmon S. (2020). TRPV1 activation stimulates NKCC1 and increases hydrostatic pressure in the mouse lens. Am. J. Physiol. Cell Physiol. 318 (5), C969–c980. 10.1152/ajpcell.00391.2019 32293931 PMC7294325

[B43] Shahidullah M. Rosales J. L. Delamere N. (2022). Activation of Piezo1 increases Na,K-ATPase-Mediated ion transport in mouse lens. *Int. J. Mol. Sci.* [Online] 23 (21), 12870. 10.3390/ijms232112870 36361659 PMC9656371

[B44] Shibasaki K. Suzuki M. Mizuno A. Tominaga M. (2007). Effects of body temperature on neural activity in the Hippocampus: Regulation of resting membrane potentials by transient receptor potential vanilloid 4. J. Neurosci. 27 (7), 1566–1575. 10.1523/jneurosci.4284-06.2007 17301165 PMC6673744

[B45] Slavi N. Rubinos C. Li L. Sellitto C. White T. W. Mathias R. (2014). Connexin 46 (Cx46) gap junctions provide a pathway for the delivery of glutathione to the lens nucleus. J. Biol. Chem. 289 (47), 32694–32702. 10.1074/jbc.M114.597898 25294879 PMC4239621

[B46] Sonkusare S. K. Bonev A. D. Ledoux J. Liedtke W. Kotlikoff M. I. Heppner T. J. (2012). Elementary Ca^2^⁺ signals through endothelial TRPV4 channels regulate vascular function. Sci. Am. Assoc. Adv. Sci. 336 (6081), 597–601. 10.1126/science.1216283 PMC3715993 22556255

[B47] Srinivas M. Costa M. Gao Y. Fort A. Fishman G. I. Spray D. C. (1999). Voltage dependence of macroscopic and unitary currents of gap junction channels formed by mouse connexin50 expressed in rat neuroblastoma cells. J. physiology 517 (3), 673–689. 10.1111/j.1469-7793.1999.0673s.x PMC2269370 10358109

[B48] Srinivas M. Kronengold J. Bukauskas F. F. Bargiello T. A. Verselis V. K. (2005). Correlative studies of gating in Cx46 and Cx50 hemichannels and gap junction channels. Biophysical J. 88 (3), 1725–1739. 10.1529/biophysj.104.054023 PMC1305229 15596513

[B49] Taruno A. (2018). ATP release channels. Int. J. Mol. Sci. 19 (3), 808. 10.3390/ijms19030808 29534490 PMC5877669

[B50] Tong J.-J. Ebihara L. (2006). Structural determinants for the differences in voltage gating of chicken Cx56 and Cx45.6 gap-junctional hemichannels. Biophysical J. 91 (6), 2142–2154. 10.1529/biophysj.106.082859 PMC1557580 16798801

[B51] Valiunas V. White T. W. (2020). Connexin43 and connexin50 channels exhibit different permeability to the second messenger inositol triphosphate. Sci. Rep. 10 (1), 8744. 10.1038/s41598-020-65761-z 32457413 PMC7251084

[B52] Veenstra R. D. Wang H. Z. Westphale E. M. Beyer E. C. (1992). Multiple connexins confer distinct regulatory and conductance properties of gap junctions in developing heart. Circulation Res. 71 (5), 1277–1283. 10.1161/01.Res.71.5.1277 1382884

[B53] Zahraei A. Guo G. Varnava K. G. Demarais N. J. Donaldson P. J. Grey A. C. (2022). Mapping glucose uptake, transport and metabolism in the bovine lens cortex. Front. Physiol. 13, 901407. 10.3389/fphys.2022.901407 35711316 PMC9194507

[B54] Zhang D. X. Gutterman D. D. (2011). Transient receptor potential channel activation and endothelium-dependent dilation in the systemic circulation. J. Cardiovasc. Pharmacol. 57 (2), 133–139. 10.1097/FJC.0b013e3181fd35d1 20881603 PMC3047599

