# Supplementary material for: Patch clamp studies on TRPV4-dependent hemichannel activation in lens epithelium
Source: Front Pharmacol. 2023 Feb 24;14:1101498. doi: 10.3389/fphar.2023.1101498 (PMC9998544; doi:10.3389/fphar.2023.1101498)

Supplemental Figure 2. (**A-B**) Individual recordings of WC Im in response to a ±100 mV ramp protocol, before (A) and right after (B) the application of TRPV4 agonist GSK1016790A. Notice the large increase of WC Im at all values of voltage explored. These experiments were pooled and averaged in Figure 2 of the main text.

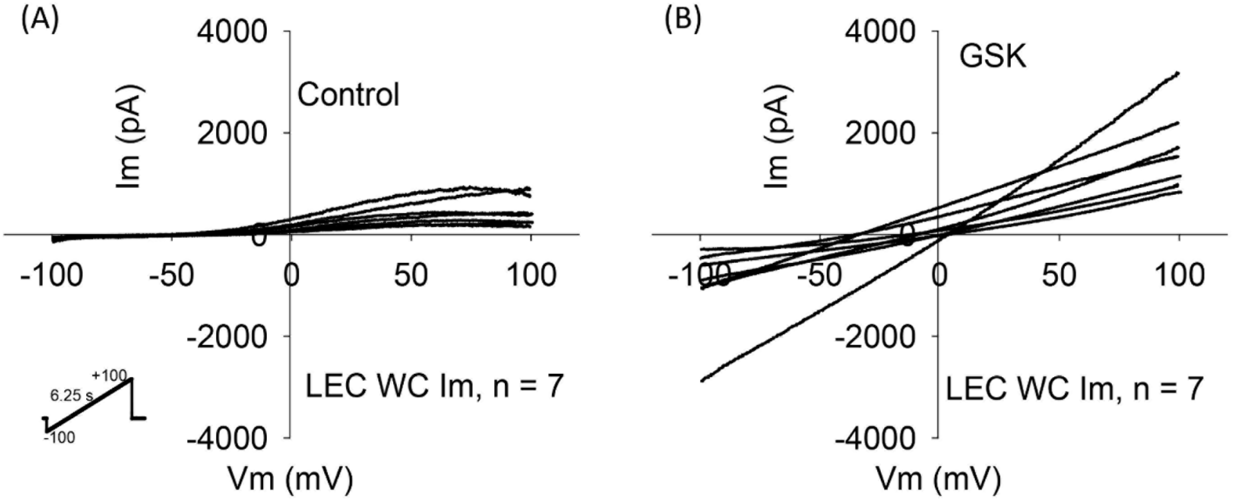

Supplement: Supplementary file 1 [file Image2.pdf]
