# Supplementary material for: Patch clamp studies on TRPV4-dependent hemichannel activation in lens epithelium
Source: Front Pharmacol. 2023 Feb 24;14:1101498. doi: 10.3389/fphar.2023.1101498 (PMC9998544; doi:10.3389/fphar.2023.1101498)

Supplemental Figure 1. (**A-B**) Individual recordings of whole cell Im at holding potential of -50 mV, from single male (A) and female (B) mouse lens epithelial cells, showing responses to the application of TRPV4 agonist GSK1016790A. Notice the variability of the response amplitude. (**C-D**) Averages of the experiments in the top panels, male (C) and female (D) derived lens epithelial cells. These results were pooled in Figure 1 of the main text.

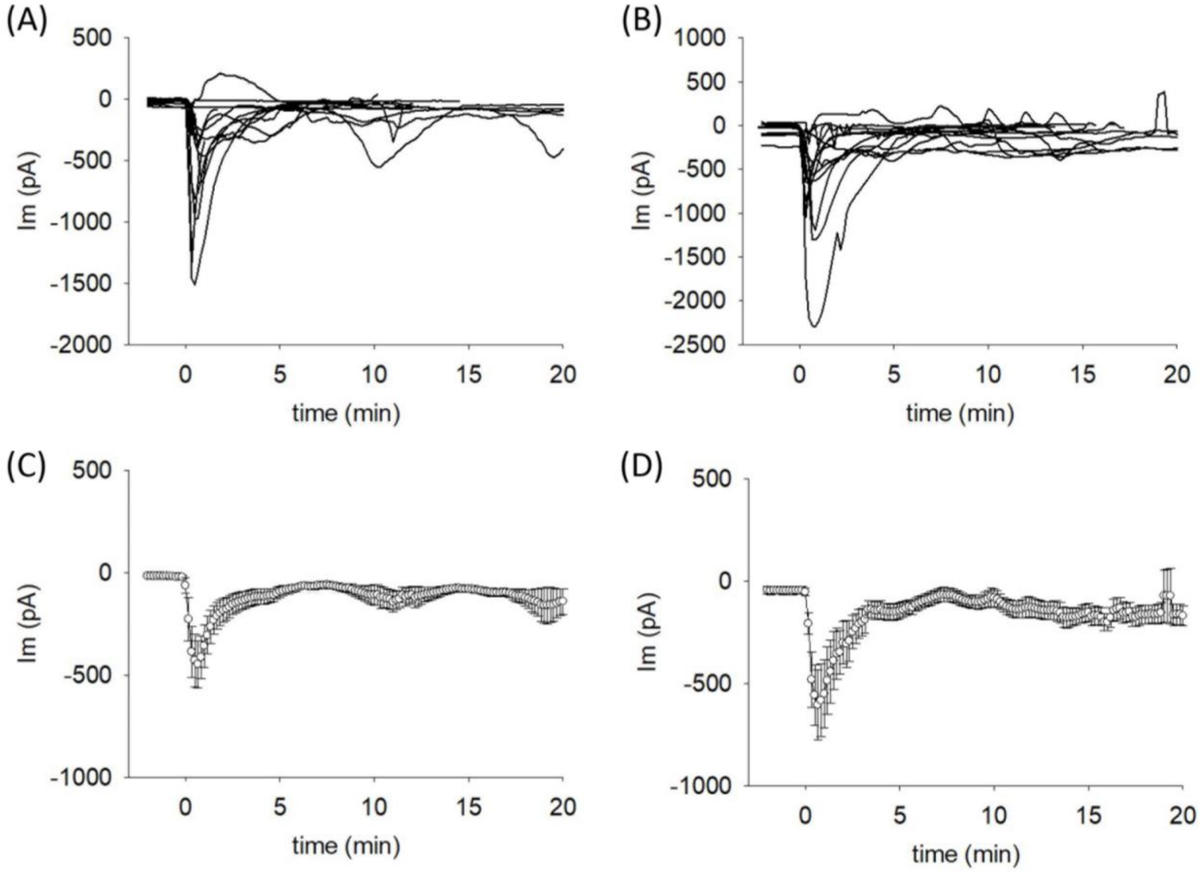

Supplement: Supplementary file 2 [file Image1.pdf]
